# Selections from the Foreign Periodicals

**Published:** 1846-07

**Authors:** 


### Periscope;

OR,

### CIRCUMSPECTIVE REVIEW.

---

### Selections from the Foreign Periodicals.

---

#### On the Use of Turpentine as a Collyrium in various Diseases of the Eye. By Dr. Laugier, Surgeon of Hôpital Beaujon.

M. Serres d'Alais having mentioned to Dr. Laugier the success he had met with in the application of the Essence of Juniper to the eye in *chronic keratitis*, with an anormal development of the corneal and conjunctival vessels, it occurred to him that *Venice Turpentine* might have a like good effect in similar cases, and he therefore gave it a trial upon several cases of acute and chronic conjunctivitis, keratitis, &c. he had under his care. All these patients were already using the *nitrate of silver* lotion, and the substitution of the turpentine was attended with marked benefit. Other cases have also been treated, and justify the author in declaring the utility and harmlessness of this remedy. He has also found it of service in opacities of the cornea. M. L. allows that his experiments with this lotion have not yet been frequent enough: but they have been sufficiently so to justify his publication of their results. After various trials he has found the following formula that best adapted for use, although some patients can bear the pure essence without any admixture.

Venice Turpentine, 20 parts,
Essence of Turpentine, 10 parts,

Place the turpentine in a marble mortar and gently heat it; and when it has become fluid, add the essence gradually. Drop three or four drops into the eye night and morning.—*Archives Generales.*

---

#### Fracture of the Lower Extremity of the Radius.

M. Blandin, in a clinical lecture upon this accident, observed that authors erroneously represent *pronation* to be impossible, while in fact it can be effected, but induces great pain. Another gratuitous assertion is, that the *interosseous space* is destroyed, when indeed it can scarcely be said to exist at all at the lower part of the fore-arm. This it is important to bear in mind, in order to avoid the injurious, or at least useless, practice of applying graduated compresses for the purpose of maintaining the space between the two bones.

The history and appearance of the case usually suffice for the diagnosis, a fall on the wrist, or rather on the palm, being the most common cause of the accident. It is, however, not unfrequently confounded with *sprain*, in which deformity of the joint from effusion also takes place. There is, however, much less mobility of the part than in fracture, and the characteristic depression of the edge of the limb (*the coup de hache*) is absent. The mere absence of crepitation does not indicate anything, as it is not often perceived in this fracture. The confounding this accident with *luxation of the wrist* has, in a great degree, arisen from the minute description given by surgical writers of this last, as if it were of common occurrence, whereas it is very rarely met with, and indeed never, except as the result of great external violence, when it is also accompanied by more or less injury of the soft parts or fracture of bones. At all events it is never produced by a simple fall on the palm of the hand.

We have placed the limb upon its ulnar side. Some surgeons only continue the splints to the lower end of the fore-arm, but if we wish to act upon both fragments we must support the hand also in the apparatus. When there is antero-posterior displacement, the mere cubital splint will not suffice, and we have seen Dupuytren much surprised at its ill-success. Compression is required at the anterior and posterior faces of the fore-arm. To supply this, we employ broad, but not graduated, compresses, merely doubling them where most pressure is required. Thus, *e. g.* if they are so folded as to present but one thickness opposite the lower fragment, and four or five thicknesses opposite the upper one in front, the disposition may be reversed behind, so as to give us a double purchase upon the broken ends of the bone. When inflammation and swelling have subsided, these may be covered with the starch bandage.

M. *Robert,* lecturing upon the same accident, observes that, like in fractures of the other pyramidal bones, the upper fragment is frequently impacted in the lower one, more or less completely. At the palmar face of the limb, near the carpus, the anterior edge of the upper fragment forms a projecting crest beneath the skin, while posteriorly there is a corresponding depression : on this account, and because of the obliquity of the fracture, the lower part of the limb much resembles the letter Z in form—furnishing a very characteristic mark of the accident. M. Velpeau represents it as a constant one : but it may be absent, until produced by the following manœuvre. Embrace the upper fragment with one hand and the lower one with the other, and exercise a certain degree of pressure at the posterior part of the arm where the fracture is supposed to be situated. This projects the fragments forwards upon the palmar surface, and exhibits the characteristic plainly. The displacement from within outwards, noticed by Dupuytren, is proved by the experience of subsequent surgeons to be a far less frequent sign than he supposed it to be.

It is important to remember that fractures of the radius consolidate very rapidly, *i. e.* in from 20 to 25 days. At this period, the apparatus should be removed and the joint exercised; for, if this be too long neglected, adhesions of some of the numerous tendons and ligaments of the part may ensue, producing a degree of stiffness or semi-anchylosis of the joint—a matter of especial consequence to a working man.—*Gazette des Hôpitaux,* Nos. 10 et 26.

---

### Fracture of the Lower End of the Fibula.

M. Robert recommends the following means of distinguishing this accident from a *sprain of the ankle-joint,* after waiting two or three days for the swelling to subside, if necessary. Apply one thumb upon the external malleolus, and the other over the supposed fracture, and transform the fibula into a lever, having its fulcrum at the inferior perineo-tibial articulation. A certain degree of pressure is to be exerted by the thumb on the malleolus externus. If the fibula is uninjured, its entire length is felt to slightly and uniformly bend under the pressure; but if there be a fracture, the lower fragment moves more or less, and projects under the finger, so that even its form may be distinguished. The fracture in these cases is almost always oblique from above downwards and from behind forwards, occurring at only a short distance from the ankle-joint.

M. R. observes that the fracture may thus always easily be detected, and is surprised the mode has not occurred to others. Dupuytren was not aware of it, but used to seize the leg with one hand and the foot with the other, which means will however only suffice to detect a fracture when situated at a considerable distance from the malleolus.—*Gazette des Hôpitaux*, No. 35.

---

STRABISMUS CURED BY AN ACCIDENTAL DISPLACEMENT OF THE PUPIL. ANÆSTHESIA OF THE IRIS AND MYOPIA OBSERVED IN THE SAME PATIENT. By Dr. TAVIGNOT.

The patient, a young girl, aged 17, and of weak constitution, was attacked by measles in 1842. From this period, the eyes, which had heretofore been healthy, became affected with strumous ophthalmia. The left one was much the worst of the two, and after 18 months of unavailing treatment, Dr. T. was consulted respecting it. He found perforation had taken place at the upper and inner part of the cornea, the iris having protruded through the corneal aperture and contracted adhesions to it. Diligent treatment by small but frequent local bleedings, purgatives, and emmenagogues, and by a great variety of local applications, eventually triumphed over the obstinacy of the disease, and restored to the patient sufficient vision for the ordinary occupations of her sex.

From her infancy she had been the subject of a double convergent strabismus, that on the right side being much the most considerable. Had her eyes not become inflamed she would have been operated upon to relieve this. Upon examining them, however, more than fifteen months after the cure of the ophthalmia, not the slightest trace of the strabismus could be discovered. The cornea of the left eye was quite transparent, except at the point where it was perforated, where there was a semi-transparent leucoma, behind which the dark, adherent, iris could be seen. The pupil was elliptical and directed from above below, and from within outwards. It acted in a normal manner, except that under a very strong light it became altogether closed. She could read small print with this eye at more than 18 inches distant, as well as when brought nearer to her. Somewhat distant objects were, however, only confusedly seen.

The appearance of the right eye was natural, and the action of the pupil normal, when the two eyes were examined simultaneously. If the left eye were closed, we observed the right pupil, after oscillating for some time, gradually enlarge and then remain motionless in a considerable, but not excessive, state of dilatation. This state continued, however bright the light, provided the left eye was kept closed, and the cornea not irritated by frictions: but energetic contraction took place the instant that light was again admitted to the left eye. When both eyes were open, it was scarcely possible to decide what part either took in the exercise of vision, for then she was enabled to read fluently, and was neither myopic or presbyopic: but when she endeavoured to read with the left eye closed, she was obliged to bring the book close to her face, and even then could not see distinctly. Believing the myopia to depend upon the dilated state of the pupil, M. T. desired her to read through a small aperture made in a card. Vision was more clear and distinct, and the myopia less distinct; but still there was a difficulty which did not exist on the opposite side.

"In this case the displacement of the pupil, upwards and inwards, has changed the relations which heretofore existed between the axes of the two eyes. The left eye, in order to direct the new pupil towards objects, is carried a little outwardly, and that the more so as being the best eye, the patient employs it the more willingly. This external deviation has produced a kind of *divergent* strabismus, balancing the convergent strabismus of the right eye, and in this way re-establishing the parallelism of the two ocular axes. We do not believe the myopia depends upon any particular condition of the humours of the eye or convexity of the cornea, but upon a diminished sensibility of the retina, (for the perforated card does not completely remedy it,) and upon a preternatural dilatation of the pupil, as the patient can read better through the card than by the unassisted eye. But we still have to inquire into the cause of this dilatation. It is evident that, for the contraction of its pupil, the right eye requires the assistance of the left, and the optic nerve, or the fifth pair, must therefore be the agent of this reflex action If it is the optic nerve of the left side which transmits the impression to the third pair of the right, which induces the contraction of the pupil, we may inquire why the optic nerve on the right side is incompetent to this, (seeing that the state of the retina proves it is not paralysed,) when in all ordinary cases one eye suffices to induce the contraction. If, on the other hand, we suppose that a kind of local paralysis exists in the sensitive ciliary nerves of the right side, we can explain the necessity of the intervention of the ciliary portion of the fifth pair of the opposite side. It is true that, in this hypothesis, we admit that the stimulus of light acts upon the fifth pair, and not upon the optic nerve, at least as far as the contraction of the iris is concerned, and that it is the fifth pair which transmits to the third the contractile movement which takes place. We consider this opinion very near the truth, and have elsewhere (*l'Experience,* 1844) exhibited the arguments which may be urged in its favour. In this case, in fact, the patient was the subject of a severe and obstinate ophthalmia for 18 months, and it is easy to imagine that the ciliary nerves spread among the affected tissues may have participated in the pathological condition, and their vitality have become consequently modified. Besides this, there existed great pain of the eyes and photophobia, so that, during the long duration of the disease, a neuralgic condition of these nerves, ending in paralysis, may have easily been produced.—*Annales d'Oculastique*, t. xv., p. 27.

---

### Midwifery Statistics.

The Reviewer in the March number of the *Archives Generales* gives the following general results of *Midwifery Statistical Tables,* recently published in the Italian and English journals. In 47,116 labours, twins occurred 446 times (9 $\frac{4}{10}$ per thousand), and triplets four times (1 in 10,000). There were 40,233 *head presentations* (969 per 1000), of which 40,046 were *vertex,* and 187 *face.* There were 1065 *breech* or *footling* presentations (27 per 1000), and 154 *transverse* ones (4 per 1000.) Of these labours, 46,632 terminated *naturally* (989 per 1000), and 484 (11 per 1000) *artificially,* viz. 321 by means of the forceps, 89 by craniotomy, 54 by turning, and 20 by vaginal or uterine hysterotomy.

---

### Case of Pelvic Abscess opened through the Rectum. By M. Amussat. Related by Dr. Compérat.

The subject of the case was a lady, 37 years of age, who had borne two children. She had enjoyed excellent health until 10 years since, when her husband infected her with gonorrhœa, from the secondary effects of which she suffered and recovered; a severe lumbar pain, however, then making its appearance, and continuing to annoy her at intervals. During the last 18 months this pain had increased in severity, and when first seen, 4th April, 1844, by Dr. Compérat, it had become very intense, occupying all the right side of the abdomen and genital organs. We need not pursue the detail of her sufferings, and of the measures which partially relieved them; but may observe that, by a recto-vaginal examination, a tumefaction was felt on the posterior part of the right side of the body.

of the uterus, seeming to be seated exactly at the recto-vaginal partition. Passing the index-finger high up into the rectum, its pulp was just able to reach the projecting part of another large swelling at the right posterior portion of the gut. All the parts were hot and irritable, and the examination gave excruciating pain. MM. Amussat and Fouquier saw the case, and believed it to be one of abscess, which the great constitutional irritation would seem to indicate, although they could not detect fluctuation, which Dr. C. believed he had felt. As the point of the finger could only just reach the swelling, the surgeons wished to defer opening it, and ordered cataplasms, the simultaneous injection of the rectum and vagina with emollients, and other palliatives. On the 19th, fluctuation had become distinct, and as the patient's general condition was now truly alarming, they determined to operate. M. Amussat having passed his finger into the rectum as high as the fluctuating tumour, slid along its palmar aspect a pair of very sharp-pointed scissors, very like those contained in dissecting cases, but having the backs of the blades rounded and the branches very much longer. The puncture was made and the blades then separated, so as to enlarge the aperture by tearing rather than cutting. During this period, an assistant pressed upon the belly, so as to project the tumour as far towards the rectum as possible. A lithotrity catheter was now substituted for the scissors, and guided along the finger, still left in the rectum. A considerable quantity of healthy, and scarcely sanguinolent pus flowed out. The cavity of the abscess was carefully injected with tepid water, and the rectum cleaned out, and the access of air prevented by ascending douches of the same. These last gave great relief. In order to keep the aperture of the abscess patent, the index-finger was introduced several times during the first day. The next day, however, it had nearly closed, and the finger could not pass through the thickness of the walls of the abscess, which M. Amussat had observed seemed like cutting into the substance of the uterus itself. He enlarged the opening by means of a longer and stronger pair of scissors, the blades of which were notched near their extremities, so as to give the instrument a lance form, and prevent its escaping during the attempt at enlarging the wound. The edges were kept separate by occasionally expanding them by means of a *brise-pierre*. The case eventually did very well.

Dr. Compérat observes that, although numerous cases of abscess forming in the cavity of the pelvis are on record (M. Bourdon gives an account of 23 of these in his paper "on fluctuating abscesses of the pelvis, and their discharge by an aperture practised in the vagina," in the *Rev. Medicale*, 1841), yet the present, as far as he knows, is the first case in which an opening has been made for the evacuation of the pus by the rectum. The surgical procedure in each case must depend upon the direction which the projecting portion of the swelling takes. Of course, the difficulties to be surmounted are less in the vaginal incision. One of these, the danger of *hæmorrhage*, was met in this case by using very pointed scissors first as a trocar, and then tearing, rather than cutting, the parts with them. The edges of the aperture also were effectually dilated by means of the *brise-pierre* as long as requisite. The introduction of instruments, however, at last induced severe *irritability of the anus*, which continued for hours, and caused the patient to dread its repetition beyond everything. M. *Recamier*, who saw the case, promised her, if she could bear very severe pain for a short period, to effectually relieve her.

"Having well oiled his fingers and thumb he formed them into the shape of a cone, and thrust them altogether through the anal ring, impressing upon the entire hand slight movements of supination and pronation in order to facilitate its introduction. The margin passed, the fingers were bent so as to increase the size of the imprisoned mass, and then drawing them out at once in this position, he forcibly dilated the sphincter, thereby causing excessive pain for some minutes. The anus spasmodically contracted during several hours, and then all became quiet. From this period, the sphincter offered no obstacle to the introduction of the finger and *brise-pierre,* and the patient suffered no pain whatever from it. I do not know whether M. R. has published his ingenious expedient; but all I can say is that, from the surprising manner in which it relieved the excessive sensibility of the anus and the anguish of the patient, it cannot be too strongly recommended in all cases where the permanent or frequent introduction of instruments irritates the sphincter."—*Revue Medicale.* t. 1, 193.

---

### On the Surgical Treatment of Croup. By M. Guersant, Jun.

I agree in the opinion of MM. Bretonneau, Trousseau, Guersant, Sen., Blache, &c. that to constitute croup the presence of false membranes in the larynx is essential. The disease may commence at the tonsils, in the bronchi, or suddenly in the larynx itself. In the first case there is more or less redness of the pharynx with swelling of the tonsils, and, what is of vast importance to notice, these last are covered with little white patches, which sometimes extend as far as the velum or uvula.

The *medical* means for treating croup are very limited. *Depletion,* once so freely employed, under the idea that the disease was a simple inflammation, is very rarely of any utility in croup, and oftentimes injurious. *Emetics* have proved far more useful as adjuvants, by favouring the detachment and expulsion of the false membranes; but alone they are not to be relied upon. *Mercurials,* especially when used early, have often exerted excellent effects upon the disease; but for these to be of service the dyspnœa must not be very urgent, or the patient very enfeebled, and when used alone they have effected but few cures.

It is upon *surgical* treatment we must usually most rely, and by it we mean the *application of caustics to the pharynx,* as well as the operation of tracheotomy. Various fluid or solid substances of this nature have been employed, but we prefer the *nitrate of silver.* Weak solutions suffice at the earliest stage of the disease, when there is little else than the pseudo-membranous deposit upon the pharynx. There are indeed cases in which these are not seen at all, the false membranes being at once deposited in the larynx, but these are rare; and frequently the membranes are not detected in the pharynx, because the first period of the disease has already passed away. At an early period the symptoms are but little urgent, and the physician, little accustomed to treat children, often neglects to examine the throat. With M. G., whenever a child manifests any febrile re-action, *such examination is an invariable rule,* and in this way he has frequently been enabled to detect the approaching disease, which would not otherwise have been suspected. He instances a case in which M. Trousseau was induced fortunately to examine the throat by observing the surface of a blister to be covered with a slight fibrinous layer—such false membranes being liable to form on the surface of any wound in those who are the subjects of diphtheritis. At first, and while the tonsils are covered with this plastic exsudation, the symptoms are not severe, so that attention is not directed to the throat. But this is a precious moment for the surgeon: for he may now frequently arrest a disease, which if allowed to go on is usually beyond his art.

While employing the solid caustic, the child should be held by a strong assistant; for it is rare that the practitioner can effect this operation unaided. The tongue must be held down by a very *large spatula,* or by the handle of a *large* spoon. If a small instrument be used you cannot effect your object. We generally employ a large wooden tongue-depressor. The caustic should, for fear of accidents, only project very slightly from its case; and many practitioners, on this account, prefer using solutions. We have already said that, at the earliest stage, the use of even a weak solution three times a day suffices. We should apply the caustic beyond the margin of the false membrane as well as to itself, as this will prevent its extension. In serious cases the solution must be very strong (1 part to 3 or 4 water), but then need only be used once daily. It may be applied by means of sponge fixed at the end of a piece of whalebone by sealing-wax. The caustic frequently dissipates the false membranes upon the amygdalæ, and yet they extend to the epiglottis. Caustic is still our best means. A larger sponge is now required, which must be fixed upon a strong whalebone, bent at an obtuse angle. The surgeon places himself on one side, and introducing the sponge right to the base of the tongue, executes some semi-rotatory movements. Sometimes the epiglottis is raised, and the fragments of false membranes detached from its inferior surface, which may be known by the paroxysm of dyspnœa this gives rise to. The caustic requires to be repeated three or four times in the twenty-four hours.

When, in spite of the energetic use of these means, success does not follow, we must have recourse to *tracheotomy.* M. Guersant has, next to M. Trousseau, performed this operation more frequently than any one else, and speaks unhesitatingly of the propriety of undertaking it, and believes that numerous failures arise from its being too long deferred. When the child will certainly die asphyxiated without, why should we hesitate to open the trachea? for cases have occurred in which the patient has lived, although false membranes have penetrated *even into the larger bronchi.* The vital point which cannot bear the slightest presence of these is the *cordæ vocales.* Practitioners who are aware that a certain number of these cases have proved successful cannot doubt the propriety of operating. M. G. usually employs a straight bistoury, and has several small sponges mounted on whalebone and a curved ring-forceps at hand. If the morbid productions do not reach into the trachea we have only to maintain the aperture patent; while, when they extend lower down, their removal may be attempted by means of the bent forceps. Among the numerous modifications of canula employed to maintain the wound open, that of M. Trousseau is an excellent one. It consists of a *double canula,* so that, when obstruction occurs, the *inner one* may be changed without disturbing that which remains in the wound. This practitioner, as well as M. Bretonneau, prior to introducing the canula, passes small sponges, moistened with a solution of nitrate of silver, into the trachea: but unless false membranes are obviously present, M. G. doubts the propriety of any such interference, and does not have recourse to it. The canula may usually be removed at the end of from eight to twelve days, but sometimes requires to be retained for 20 or 30.

The air of the chamber should not be kept too dry and hot. To render it sufficiently humid it is a good practice to evaporate some emollient decoctions in the room several times a day. It is a difficult thing to maintain an equable temperature about the child, but for a long period I have experienced the utility of wrapping around the neck, without tightening it, a light woollen comforter having its meshes very widely knitted. By this contrivance, the air, before it reaches the trachea, becomes sufficiently warmed. When the canula becomes obstructed, the inner canula should be removed and cleansed, instead of thrusting sponges into it, which may only increase the obstruction. When it is deemed proper to cleanse out the trachea, only the most delicate whalebones must be employed. When indurated concretions form, both canulæ should be removed and the patient encouraged to expel them by coughing. I have never removed the canula before the tenth day, but M. Trousseau has done so on the third or fourth. He advises us not to remove it suddenly, but for one, and then for several, hours daily.

Finally, let us observe that croup is so grave and so constantly mortal a disease, that we should frequently have recourse to this operation before it reaches its last stage. "While tracheotomy was almost always a powerless weapon in my hands," says M. Trousseau, "I always recommended its performance as late as possible; but now I have met with numerous instances of success, I always say it should be performed as early as possible, as soon in fact as no other chance of success remains." Of 136 children operated upon, M. T. has saved the lives of 32; and, without being so fortunate as that practitioner, in 36 cases I have met with four successful ones—a success sufficiently great for us to lay it down as a law that we should interfere rather than allow the infant inevitably to die.—*Gazette des Hôpitaux,* Nos. 48 and 52.

(The practice of promptly examining and cauterizing the pharynx may doubtless prevent the extension of the disease in some cases, and is not enough attended to in this country. So, also, an unreasonable prejudice against *Tracheotomy* prevails among us. Death stares us in the face, and why not resort to means which has rescued many lives, albeit these may be few compared to the numbers operated upon—many of whom were *in extremis.* Dr. G. does not notice the external application of heat (to rubefaction) so useful at the outset of croup).—*Rev.*

---

### Phosphate of Ammonia in Gout and Rheumatism. By Dr. Buckler, Baltimore.

Dr. Buckler thus states the grounds upon which he recommends this substance to notice.

"Taking into account these two prominent facts above stated, namely, the excess of lithic acid found in the urine at the period of convalescence from an attack of acute gout or rheumatism, and the subsequent deposit of soda and lime in the white tissues, it occurred to me that, during the existence of these diseases, the lithic acid might exist in the blood in a state of combination with soda and lime, in the form of insoluble compounds, which the kidneys and skin refuse to eliminate. If then, any agent could be found capable of decomposing the lithates existing in the blood, and of forming in their stead two soluble salts, which would be voided by the kidneys and skin, we should thereby get rid of the excess of fibrin in the blood, the symptomatic fever and the gouty or rheumatic inflammation, wherever seated, which have been excited by the presence of these insoluble salts. It occurred to me that *phosphate of ammonia* might be the agent, provided it could be given in doses sufficient to answer the end without producing any unpleasant physiological symptoms. If our theory were true, phosphate of ammonia seemed to be the proper re-agent, for it would form, in place of the insoluble lithate of soda, two soluble salts, the phosphate of soda which is remarkably soluble, and the lithate of ammonia, which is also soluble, and both capable of being readily passed by the skin and kidneys. The excess of uric acid would thus be got rid of in the form of lithate of ammonia; and the soda, floating in the round of the circulation (instead of being deposited, as it were, like an alluvial formation in the substance of the fibrous and cartilaginous tissues) would be taken up by the phosphoric acid, and eliminated from the circulation."

The particulars of thirteen cases of gout and acute rheumatism are briefly related, in which this medicine (*in doses of from ten to twelve grs. ter in water*), seems to have wrought a speedy cure. Dr. B. has also found it an excellent remedy in old hospital chronic cases, which had resisted iodine and other medicines; and in those instances in which the deposits and deformities are such as to be irremediable, the acute attacks which supervene may be effectually relieved. "In reviewing the cases which I have published, it will be noticed that thickening of the white tissues, of long standing, has disappeared under the use of the phosphate. Now, it is in such cases that the lithic acid diathesis generally prevails, and this agent seems to act here by depriving the blood for a long time of uric acid and soda, thus creating a demand for these elements in that fluid, and thereby bringing about an absorption, as it were, and solution of the superfluous lithate of soda which is deposited in the white tissues." In all the cases adduced, when lithic acid had been before observed in the urine, it disappeared under the use of the phosphate, while, instead of the lateritious deposit usually seen at the period of convalescence, this fluid continued limpid—the eliminated lithate of ammonia and phosphate of soda being soluble. The rapid disappearance of lithic acid from the urine in cases wherein this salt is administered, leads to the conclusion that it must prove an eligible remedy for *uric acid calculus.*

Dr. Buckler does not propose the salt as an exclusive and empirical remedy, but employs simultaneously, various means for combatting serious symptoms, until the healthy condition of the blood is restored.—*American Journal Medical Sciences*, Jan.

---

### On the Submucous Division of the Sphincter in Affections of the Anus. By M. Demarquay.

The division of the sphincter, as practised by Boyer, frequently fails, and is not devoid of danger; but all its advantages may be obtained and inconveniences obviated by operating upon it beneath the mucous membrane. MM. Guerin, Velpeau, Blandin, and the author, have employed this modification with great success, and it is the object of this paper to spread a wider knowledge of the proceeding by detailing several of the cases which have been treated by the two last.

Submucous myotomy is especially calculated for the relief of *spasmodic action and contraction of the sphincter ani.* It is often difficult to say where one of these affections ends and the other begins, for they are so intimately connected that the one may be said to be a consequence of the other: for if the same irritating causes which induce spasmodic contraction of the muscle continue in operation, this will then become permanent. When the contraction is well-marked the anus seems deeper-seated than ordinary, the borders of the external sphincter being seen projecting and the pleats of the region well-marked. If hæmorrhoids or mucous membrane has become engaged within the sphincter, the compression gives them a violet colour, and they may even become gangrenous. The finger is introduced with difficulty, and with pain to the patient, and is much constricted by the muscle.

Spasmodic contraction, requiring the operation, may manifest itself under various circumstances, but it is especially in its prevention of the *extraction of foreign bodies* from the rectum, that it comes under our notice. It may also manifest itself in two very important affections, viz. *prolapsus of the rectum* and the *sudden appearance of large hæmorrhoids.* In this last case, the constriction may be so considerable as to induce gangrene, or at all events prevent the return of the protruded parts.

Contraction of the sphincter plays an important part in three affections, viz. constipation, old hæmorrhoids, and fissure of the anus.

(1.) Prolonged and obstinate *constipation* not unfrequently gives rise to it. The indurated matters contained in the gut irritate it, and induce, first spasmodic and then more permanent, contraction; and authors have certainly sometimes mistaken the effect for the cause, in regarding the constipation as produced by the contraction. In mentioning constipation as a cause of contraction, it is not intended to recommend combating it by a surgical operation. It is a complex condition of which this contraction only forms a portion.

(2.) When *hæmorrhoids* have long existed, and become frequently congested and inflamed, it is by no means rare to find contraction induced. In this case they are forced out at stool, but can only be partially returned. They inflame; defæcation becomes more and more difficult, and fissures and hæmorrhages result. Several examples are given in which this state of things was effectually relieved by the submucous section.

(3.) *Fissure of the Anus* is so intimately connected with contraction of the sphincter that Boyer regarded them as the same disease. But we have already seen contraction may exist without fissure, and the question is, when the two affections co-exist, which has preceded the other. In the great majority of cases a more or less obstinate constipation, a state often giving rise to contraction, has preceded the fissure. Authors, however, have stated that they have observed fissure without contraction, and if they are correct we may explain such cases by the varieties of the disease admitted by M. Blandin. He states that the fissure may be seated above, opposite, or below the sphincter. In the last case it may exist without contraction.

The author has analysed all the cases of fissure of the anus which have been published, as far as he knows, since the time of Boyer. They amount to 53, of several of which very insufficient particulars are given. Of these persons, 30 were men and 23 women, in opposition to Boyer's assertion that, females are most liable to the disease. In six instances the fissure was double, triple, or quadruple. The age is only recorded in 42 cases, the disease being most common between 20 and 30 and 30 and 40. In 26 of the cases constipation, and in six cases hæmorrhoids, are described as existing.

Seven cases of fissure operated upon by M. Blandin are given, in all of which complete and immediate relief succeeded to the most intense suffering. Prior to commencing the operation the rectum should be well-cleared out, so as to obviate the necessity of going to stool shortly after it. An assistant must raise the side of the buttocks opposite to that on which the section is about to be made. This last is always practised at one of the sides of the anus, so that the sphincter may be divided through its middle. An ordinary *tenotome* may be used, or a bistoury which M. Blandin has contrived. The former is however not long enough, and its point is not sufficiently guarded, so that there is danger of penetrating the mucous membrane with it. M. Blandin's bistoury is protected by a moveable sheath, and he can either use it for puncture or incision, or as a flattened probe, accordingly as he wishes to divide parts, or merely to pass it between the muscle and mucous membrane. Whichever may be selected, the operation is simple. 1. Make a small opening in the skin. 2. Introduce the finger into the rectum, at the same time that the skin on each side of the anus is stretched. 3. Pass the tenotome between the mucous membrane and the sphincter. 4. Divide the latter. The puncture of the skin is most conveniently made at from 8 to 12 lines from the anus. The instrument must be passed in very gently so as to detach parts as little as possible. When the division has been made a kind of crack is heard, and the finger which is in the rectum feels the space between the two divided portions of the muscle. Although not absolutely necessary, the patient had better keep his bed for a few days after; and he should live very abstemiously, as it is desirable he should not go to stool for three or four days, after which there is no fear of rupture of the cicatrix. Occasionally the section of the sphincter on one side only is insufficient. The fissure does not after the section of the muscle require any special treatment.—*Archives Generales*, April.

(When the dreadful and prolonged suffering incident to fissure of the anus is considered, the substitution of this simple operation for that of Boyer's must be regarded as of one the most valuable applications of myotomy.—*Rev.*)

### Quinine in Acute Rheumatism.

Three years have elapsed since M. *Briquet* communicated the success which attended the use of quinine in cases of acute articular rheumatism at the Hôpital Cochin. Several practitioners have resorted to it with the same result: but its employment has become by no means general, partly in consequence of too large doses being given at first, and various accidents in consequence ensuing. M. Briquet formerly gave from 4 to 6 scruples in the 24 hours, but now gives but from 1 to 4, discontinuing it when any sign of prostration manifests itself. He employs the neutral salt rendered soluble by sulphuric acid. From the first or second night sleeplessness disappears, and a little later there is a more or less marked diminution of the pain and swelling of the joints. From the third to the sixth day the rheumatism may become cured; but when the cure is so prompt as this there is usually some return of pain, with or without swelling, again requiring the use of quinine. As a general rule the patients are cured or notably relieved from the ninth to the twelfth day of treatment.—*Gazette Medico-Chirurgicale*, No. 17.

---

### Contagiousness of Puerperal Fever.

Dr. *Kneeland* has contributed an interesting paper upon this subject to the *American Journal of Medical Sciences*. He does not bring forward any new cases in elucidation of the question; but, from a careful examination of facts and authorities on both sides of the Atlantic, arrives at the following conclusions.

"1. From the confinement of cases to the practice of single physicians and nurses in populous cities; from the fatal results attending post-mortem examinations; from its ravages in hospitals; that puerperal fever is contagious; that it may have other modes of propagation in certain states of the atmosphere, and among strongly predisposed individuals; but that the fact of its conveyance by practitioners attests its contagiousness. 2. That it may be propagated by direct inoculation with the fluids of the living and dead; by the effluvia arising from the bodies of the sick, inhaled in the very chamber of death (as in the wards of a hospital) or carried about by the person of the physician; by clothes, bedding, which have been in contact with a diseased individual. 3. That the order of propagation from the physician to the patient, and the regular succession of cases, show that the epidemics of puerperal fever are, in almost all cases, the *effects* and not the *causes* of contagion. 4. The contagion acts according to the frequency of communication between the physician and nurse (in whose practice are cases) and lying-in-women, independently of insalubrity of places, wretchedness of patients, or the neighbourhood of dwellings—for, although poverty and misery seem to predispose to it, communication is none the less fatal to the higher classes. 5. A case, to all appearance sporadic, may communicate the disease; a mild case may communicate a severe disease, and *vice versâ*. 6. Immunity proves nothing against contagion; it may be the effect of an acquired or temporary inaptitude. It is equally inexplicable in all contagious diseases. 7. The rapidity of its propagation shows that it is contagious at the commencement: the fatal results of attending autopsies indicate this character after death. 8. That a physician should not make or be present at an autopsy of this disease; or if he does, should take proper measures to cleanse himself and dress, for the safety of his next patient—that if a case (or several cases) occur in his practice, he should consider himself, in the language of Dr. Holmes, a 'private pestilence,' and regulate his conduct accordingly—that persons who have washed, or have otherwise handled the clothes or bedding soiled by the discharges of this disease, should not approach, much less nurse, a woman after delivery. 9. That when the disease is prevalent, a prompt removal from possible intercourse with a 'pestilential' physician, and a strict attention to ventilation, cleanliness, quiet, proper food, &c., are the dictates of a reasonable fear."

(So overwhelming is the testimony in favour of the contagious character of this disease, and so truly frightful have been the consequences which have frequently resulted from neglecting the precautions this should have furnished, that we think a medical man persisting in attending successive cases of midwifery after a well-marked instance has broken out in his practice, should be made amenable to the criminal tribunals of his country. A woman has confided her life and safety to his skill and honour, and he enters her chamber, and approaches her person, the bearer of what he knows is reputed to be by some of the wisest of his profession a deadly poison, the escaping from the effects of which she is to owe to her own idiosyncracy and not to his humanity. Medical men are doubtless unconsciously the propagators of other diseases; but their instrumentality is here so well marked, and has been so often exemplified, and the consequences are so commonly fatal, that every one possessing a spark of conscience should at once resolve to relieve himself from this dreadful responsibility, at whatever sacrifice, while such as choose to persist in so criminal a course should not be allowed to do so with impunity. This is not one of the hundreds of instances of difference of opinion in the profession where each person is as likely to be as right in the long run as the other. The practical results are fearful, and it is of the last importance for the honour of our profession that we do not convert our blessed ministration into a curse and a scourge. It is to be regretted that an authoritative and combined declaration of opinion has not been expressed upon the subject, and a rule of proceeding laid down; for we every now and then hear of men consulting their brethren and temporarily abandoning their practice only *after* they have lost several patients in succession; and Dr. Jackson of Philadelphia, our author states in his paper, *began* to be "seriously alarmed on the score of contagion," when he had had seven cases of puerperal fever in uninterrupted and rapid succession (five of which were fatal), and women whom he had been engaged to attend fled from as from a pest. Mere ablution or change of dress will not suffice. Midwifery should be entirely abandoned for several months. The sacrifice would sometimes be great, but it would not be without its compensation.—*Rev.*)

---

### On the Question of the Removal of Cancerous Breasts. By M. Velpeau.

The patient before us is one upon whom we cannot operate, for, on examining the breast, we find little knots extending even to the edge of the great pectoral; moreover the nipple is implicated, and it is an instance of disseminated cancer, which for us is a true *noli me tangere.* We do not mean to say that the wound would not cicatrize and the patient recover of the operation itself, but we have acquired the desolating conviction that this species of cancer, as well as the ligneous scirrhus, invariably relapses.

Many descriptions of cancer should not be removed, and this is a very important point, as many practitioners, unaware of it, injudiciously undertake an operation. Among the varieties liable to return, all do not re-appear however with the same tenacity. Among those whose return is certain may be named that form in which the skin becomes horny or leathery, and in which the indurated integuments and the nipple are retracted; that ligneous variety in which the indurated parts are formed into layers like bark; and that in which the disease does not commence with one tubercle under the skin but with several at once, in which case we may be certain that there are others there also. In all these, relapse is certain. In this last-named variety we need not be surprised at a return, for how are we to be certain of the removal of cancerous grains so small as to elude sight and touch.

*Melenic* cancer is rare in the breast, or co-exists in other parts of the body. Nevertheless, if a simple tumour of this kind appears in this part, it should be removed, as it is not always reproduced. *Colloid* cancer may be placed in nearly the same category. *Scirrhus* also admits of operation, except when it is branching, *i. e.* sends out roots in different directions, whence its name *cancer.* In this case it is usually reproduced, its distinct limits being ascertained with difficulty. *Encephaloid* is a bad species, because it is usually simultaneously developed in one or several other organs. If, as far as we can ascertain, no other deposition exists, we have a chance of success by operation. We have cured some fifteen out of those we have operated upon; but when we recollect that these tumours may become developed in some internal organ without any symptom thereof, we can see how uncertain any prognosis must be. There are some curious facts connected with this subject. I recollect a man of robust habit and apparently in vigorous health, from whose lip I removed an encephaloid tumour. He died in four days, and at the autopsy we found thousands of little encephaloid tumours in the liver. Are we to suppose these became developed in so short a period after the operation? If we believe that they existed simultaneously with the disease of the lip, how do we explain the excellent health of a person having so large a quantity of cancerous tumours in his liver?

It will be seen that cancerous tumours in general, and those of the breast in particular, demand classification and distinction from each other; and until classification is perfectly established, and the same one employed by all practitioners, they will continue to misunderstand each other.—*Gazette des Hôpitaux,* No. 55.

This excellent surgeon published some interesting observations in the same journal last year upon *Neuralgia of the Breast,* so frequently mistaken by women for cancer of the organ. If the surgeon is not on his guard when he examines the breast, he observes, he may confirm instead of dissipating the error. The organ is composed of numerous lobules which are far from always offering the same consistence. In different women, too, there is great variety in the configuration of the thorax as regards its flatness or projection, which imparts a different sensation to the hand engaged in examining the mamma. Again, if we take hold of the breast at the side as if to compress it, and then feel it with the other hand, it will be difficult to persuade one self it does not contain a tumour: and without having made the experiment the different density of the organ, according as it is pressed in one direction or another, cannot be conceived. If to this we add that the gland is more or less developed, and the breast more or less resisting, more or less pendent, in different persons, we can see there are many shades of difference met with.

Women who suffer severe pain always believe that cancer exists, and have submitted themselves unnecessarily to amputations and severe treatment. Attacked by a species of hypochondriasm, they are annoyed if you doubt the importance of their malady; and although you may deny that they suffer from the disease they suppose, it would be unjust and inhuman to forget they do suffer much. The pains felt are sometimes only the result of the attention being unduly directed to unpleasant sensations and the mind dwelling upon them; but at others result from a true neuralgic affection of the breast. It is of course important to distinguish this from cancer, and we must remember that in general true tumours do not cause pain at their commencement, and that, when the patient has long complained of violent pain without any tumour being apparent, there is no tumour at all in the case. In examining the breast we must never take hold of it by its side, but on the contrary expand it by means of the entire hand; and take every opportunity of comparing the sensations derived from the lobules of this organ with those produced by pathological tumours. When, after believing we feel a tumour by pressing it laterally, we are unable to do so by pressing from before backwards, none in fact exists.

The means of treating this affection consists in part in modifying the form of the stays worn by the woman. The breast must be supported. As the fashion now leads to the breasts being widely separated from each other, the inner side often suffers pain from the traction exercised. The stays should be so made as to support the breasts towards the median line, bringing them nearer to each other, and carefully avoiding compressing them. This precaution alone often suffices, but we may also employ various liniments; and give iron, bismuth, valerian, &c. internally, according to the condition of the patient. It is not, however, in women of high-life alone that we observe this affection, but examples are often met with among those of the lower classes.

---

### General History of the Seven Diatheses. By Professor Gaillard.

By *diathesis* we understand a pathological modification which exhibits itself in affections variable as to their seat, but identical in their nature. It is a change in the organs and functions common to the entire economy; and when the morbid state is not so general as this, it affects at least some of the elements which enter into the composition of many organs. Thus rheumatism is seated in the fibrous tissue, and the herpetic diathesis occupies the skin and mucous membranes. Affections engendered by this cause put on special forms in relation to the diathesis whence they have arisen. Thus common ophthalmia is distinguished from syphilitic, and this from herpetic and scrofulous ophthalmia.

A diathesis is more than a mere simple *disposition to a disease,* and a person under its influence may be in a very serious condition long before any local symptom manifests itself. A person from whom a scirrhous tumour has been removed may remain for a long period without a relapse, and yet the cancerous matter eventually become deposited in some other part. It as it were hesitates, and if some external agent produces any local stimulation, it attacks this irritated part by preference.

Many persons look upon every disease as a mere *local lesion,* as bronchitis, arthritis, gastritis, &c. to which all medical appliances should be addressed; but the situation and extent of the anatomical lesion should not attract our attention so much as the *general cause,* which may give rise to a similar lesion in some other part of the economy. In this way a sprain complicated with scrofula may be allowed to degenerate into a white-swelling, or slight gastric irritation, under the rheumatic diathesis, may become developed into an obstinate gastralgia.

The diatheses constitute the *humoral vitiations* of the ancients, but they are not *cachexiæ.* A cachexia is a general condition of decay, a bad state of the general health usually consecutive to some serious or organic change. Certain diatheses may give rise to cachexia, but this term could not in general be applied to a gouty or rheumatic subject.

We must also distinguish a diathesis from the susceptibility to morbid causes, the *vulnerability,* which certain organs or apparatus may furnish. The vitality of the different organs may vary either above or below the normal mean, and the susceptibility in either case be increased. Such vulnerability may depend upon (1) congenital disposition: (2) hygienic influences: (3) certain endemic or epidemic medical constitutions: (4) antecedent disease.

Considered in a general point of view the *diatheses offer these characters in*

*common.* 1. They act on several organs or regions simultaneously or successively. 2. They remain latent for a certain period, and then suddenly manifest themselves by various local lesions. 3. They perpetuate acute affections, interrupt their natural course, prevent the resolution of the disease, and lead to a chronic condition. 4. They obstinately persist, unless attacked by general measures. 5. They give rise to frequent relapses.

There are certain organs and tissues which each *diathesis especially* affects: thus rheumatism affects the fibrous tissue, syphilis the skin and mucous orifices, &c. But as these anatomical elements are common to many regions, the distinctive character of the diathesis is still maintained. That is, it produces at distant points local manifestations which are so identical that they alternate with, succeed, or displace each other, furnish analogous physical and physiological signs, and are modified by the same means of treatment.

Some diatheses are *fixed or adherent* to the organs which they attack. They may be met with at several points, but they leave with difficulty organs they have once attacked. This is the case with scrofulous, cancerous, and syphilitic affections, while others are *mobile*, and change their site with singular rapidity. This is the case with the rheumatic and gouty, and, in a less degree, the herpetic diathesis.

These affections are not all produced by the same *causes*. Thus, scrofula and scorbutus arise from the exertion of a profound and lengthened modification of the solids and fluids of the economy by external causes. Syphilis and cancer are produced by a poisonous principle, a material and organic cause, circulating in the blood. Rheumatism, gout and herpes may also be explained upon this last principle. Diatheses are often *hereditary*, *i. e.* the new-born child possesses a particular vulnerability in certain systems, and an especial susceptibility in regard to certain modifying agents of the economy.

The *prognosis* of diatheses varies according to the nature of the diathesis, the intensity, seat, and duration of the local disease, and the social position of the patient. This last is of great practical importance; for when the condition of life of the patient places at his disposal the resources of hygiene and therapeutics, and his habits are temperate, the physician is much more assured of the results. He may then attack the most obstinate disease, and impress modifications upon the economy which meet it at its point of departure. Still, it must be allowed, that the favoured classes of society are more delicate and susceptible, and less amenable to the employment of medicines. Chronicity and obstinacy are characteristics of the diathesis. The general condition is modified with difficulty. The means employed are tedious and insensible in their action. A diathesis is slow in forming and in disappearing.

Diatheses are often *complicated with each other*. A patient, suffering from the herpetic diathesis may contract syphilis and rheumatism, and manifest symptoms characteristic of these different causes. In general the phenomena are characteristic, but sometimes the diagnosis is rendered difficult. Rheumatic and syphilitic pains may be confounded. Herpes of the prepuce has been mistaken for chancre. M. G. believes, with many other authors, that rheumatism may supplant syphilis, and that it is an error to treat all articular swellings and pains which follow blenorrhagia as venereal.

The *treatment* must, above all things, be general, and adapted to the nature of the diathesis. It comprehends—1. Special modifiers, or to speak more exactly, such as are oftener employed with usefulness and efficacy in each diathesis. 2. Evacuants, as purgatives, sudorifics, blisters, issues, &c. These last seem to replace the pathological local fluxion induced by the diathesis, and constitute, as it were, a supplementary function. 3. Narcotics which modify the nervous system and vitality of organs. 4. Hygienic modifiers. These are the most efficacious. With them we may frequently do without medicines, but nothing will replace them.

The diatheses are *seven* in number, viz. the *scrofulous, scorbutic, herpetic, syphilitic, rheumatic, gouty,* and the *cancerous.* Each of these, to be properly treated, would require a special article, but we can only notice one of the examples adduced by the author. A *simple sprain* is always cured, quickly or slowly according to the prudence of the patient, but still it always is cured; and it is only under the influence of a diathesis it becomes a grave disease. "In the majority of instances," says Lisfranc, "the external violence is only a determining cause which fixes in the articulation a morbid principle existing in the economy."

The *diagnosis* of diathesic diseases is established by several circumstances. 1. The disease is chronic and obstinately persisting. 2. The patient has already been the subject of rheumatic pains, herpetic eruptions, glandular swellings, or some other affections which announce the existence of a diathesis. 3. In the origin of the actual disease, the patient has been exposed to circumstances which radically modify the economy. Thus the prolonged action of cold and damp engenders rheumatism. 4. The local manifestations offer special characters which indicate the generative cause. 5. In cases of doubt we test the disease by special medications, and the advantageous or injurious results which ensue throw light upon its nature. A young lady, the subject of subcutaneous swellings and gnawing ulcers, which offered many of the characters of syphilis, was treated with Dupuytren's pills without advantage, but recovered shortly under the influence of bark, as the lesion was caused by a scrofulous diathesis. 6. If the antecedents enlighten us upon the existence of a diathesis, consecutive phenomena often demonstrate the reality of certain causes, which had been either only suspected or even misunderstood. A person labouring under gastralgia is seized with a pain in his shoulder, and the affection of the stomach ceases. A dry and irritating cough may disappear as soon as an eczema of the thigh manifests itself. Sometimes these diathesic symptoms appe[illegible] several years after the cure of the disease, and explain to us its capricious progres[illegible], its unusual characters, and its obstinacy.

In fine, many, or rather the great majority of, chronic diseases are maintained by general causes, and it is to these we should address our principal medical appliances.

---

### New Means of Disinfecting Dissecting Rooms.

M. Sucquet has presented the Academy of Sciences with an account of his new mode of impeding the putrefaction of subjects used for dissection. He observes, the means which at present imperfectly accomplish that object, as arsenic, corrosive sublimate, &c., either injure the edges of cutting instruments, or prove detrimental to the health of those who use them. He employs two substances, namely, a solution of *sulphite of soda* and one of *chloride (chlorure) of zinc.* The first of these is injected into any large artery, returns by the veins, and even fills the lymphatics. It exerts no chemical effect upon the blood, but effectually cleanses out the minutest capillaries, so that other injections may be used afterwards. The volume, consistence, colour, &c. of parts are all preserved, and remain so, if the integument be undisturbed, so that air does not obtain access, for a month as an average period, but sometimes even for 40 or 50 days, and that in a moist atmosphere at a temperature of 54°. The sulphite absorbs the oxygen given off by the tissues, and prevents it taking part in the putrefactive process.

This substance, however, does not protect from putrefaction for more than 20 days, when the integument is destroyed and the tissues exposed to the air; but the parts may be preserved for ever free from putrefaction by the use of the *chloride of zinc.* Parts required for further dissection should be immersed in it:

and all those in which putrefaction is about to commence should be daily sponged with it. Its action is instantaneous. Parts which have become green and softened are at once disinfected, all the tissues it comes in contact with becoming white and hard. It acts by precipitating the soluble portion of the animal fluids, as albumen, fibrin, &c., and it coagulates cerebral and fibrous tissue.

Both substances are expensive to purchase, but their preparation on a large scale, the formulæ for which the author supplies, may be conducted very economically; so that three or four shillings will defray the expenses of preserving each subject.—*Archives d'Anatomie,* 1846, p. 122.

(In the *Annales d'Hygiene* for April is a short paper by M. Guerard, who was one of the Commissioners appointed to examine into the success of the experiments with these substances which were carried on during several months at the *Ecole Pratique.* The field is a wide one, for during 1845, there were upwards of 1500 bodies received for dissection. At *Clamart,* where the process is also about to be adopted, the average exceeds 2800: so that the annual number of bodies dissected at Paris considerably exceeds 4000—an enormous number when compared with that furnished to the London Schools, where the difficulty in obtaining subjects seems to be annually increasing, and the supply of which is entirely inadequate for the proper teaching of anatomy. The more limited the supply, however, the more important the means here proposed for economizing it, providing it prove as efficacious as represented. M. Guerard's statement is most unequivocally favorable to it. He states that he has repeatedly visited the dissecting-rooms since it has been in use, and finds them utterly devoid of all offensive odours, and the complaints which used to be made on account of these, by the inhabitants of the locality, have entirely ceased. Bodies may be dissected for from 15 to 40 days, according to circumstances, without any attendant inconvenience.—*Rev.*)

---

### On Typhoid Fever. By M. Bricheteau.

M. Bricheteau has recently published some interesting papers upon this subject, of which our limits, however, forbid any prolonged notice. He maintains that the history of the disease proves that, forty years since, the intestinal canal did not present the lesions now so commonly remarked, for these must have been noted by such observers as Bayle, Dupuytren and Laennec. It may be laid down as a principle, therefore, that *typhoid fevers, without absolutely changing their nature, present at certain epochs notable modifications sufficient to effect a change in their diagnosis and treatment.* An illustration of this is also derived from the fact of no such considerable changes being observed in the epidemic fevers of Great Britain and Ireland. Even in France such lesions are absent in some epidemics, and are in others no wise in proportion to the gravity of the symptoms; so that they cannot be considered the essential feature of the disease and its point of departure, although, from their dangerous character, they naturally have excited much attention.

M. Bricheteau observes that the contagious character of typhoid is not admitted in Paris, although believed in in some of the provinces; but he is certainly in error in stating that the contagion of typhus is generally admitted in this country, and that a vigorous sequestration is consequently insisted upon. He is more correct as to the treatment which prevails here, for certainly the bleeding and antiphlogistics employed by many in Paris, would meet with few approvers in London. In explanation, he observes that inflammatory lesions are more common in Paris, as its climate is less debilitating than that of London. Sydenham, who had studied disease at Montpellier, acknowledged that his practice was too vigorous at the commencement of his career in London; and many other practitioners have oberved that bleeding is much less easily borne in that capital than in Paris.

M. Bricheteau believes, however, that typhoid cannot be looked upon as a *phlegmasia*, inasmuch as neither the season of the year at which it occurs, the treatment it requires, the order of sequence of the abdominal symptoms, or the measures which relieve these, favour this view. "By seizing on this inflammation as a basis of treatment, we commit the error of neglecting the cause for the effects of the disease." M. *Delarroque*, of the *Hôpital Necker*, and others, believe the symptoms of typhoid are due to the irritating effect of *vitiated bile and other secretions* which are thrown out upon the intestinal mucous membrane, and are then absorbed, poisoning the economy. Andral, Bouillaud, and Louis, are cited in proof of the enormous quantities of such secretions which are found after death, while the success of the purgative treatment pursued by M. D. is adduced in corroboration of this view. Other observers explain all the symptoms of typhoid by a primary *alteration in the condition of the blood*. The dissolved state of this fluid has been often observed by both the older and modern practitioners, and symptoms identical with those of typhoid have been induced by injecting putrid matters into the veins—the blood losing its plasticity as in typhus.

*Conclusions.*—The placing the seat of typhoid in the intestinal canal, the cerebro-spinal system, the blood or its secretions, seems to me of very little consequence, since this disease affects simultaneously or successively every portion of the economy. We find first, the phenomena incidental to a disturbance of the nervous system, in which disturbance the circulation soon participates. Almost immediately afterwards, diarrhœa and other symptoms of intestinal irritation present themselves, as also the *râle* which proves the pulmonary organs are not unaffected. Other symptoms soon show that the various secretions are either perverted or temporarily suspended. The affection seems to prevail in some degree everywhere, and to have its special seat nowhere. It is truly the *morbus totius substantiæ* of the old physicians. It is often epidemic, and sometimes contagious, presenting much analogy with many pestilential fevers, typhus, sweating-sickness, and intense epidemic influenza—diseases to none of which a precise seat can be attributed. It follows that the *treatment* of typhoid should be mixed, being composed of measures as various as the lesions to be combated. Sometimes diluents and antiphlogistics, sometimes evacuants, at others alteratives, tonics, antiseptics, or sedatives may be required. In fact, to adopt any exclusively curative method, would be to apply a determinate medication to an unknown pathological condition.—*Gaz. Med. Chir., Nos.* 13, 15, 17 & 23.

---

### English and French Medical Periodicals.

The *Gazette Medicale* for June 13th publishes an article upon the English Medical Periodicals. That it is replete with error will not excite the surprise of those who are aware how completely everything relating to this country is misunderstood in France, and who recollect the ludicrous mis-statements of M. Malgaigne (*Med. Chir. Rev. Jan.* p. 284), at the Medical Congress, in reference to the position and acquirements of our general practitioners. Still, writers who profess the instruction of their readers upon any point, should at least inform themselves of the facts, and we regret our space will only permit our indicating one or two instances in which the present one has disregarded these. He states that the Quarterly Medical Reviewers of this country confine themselves to the insertion of mere *verbatim* extracts from the works they examine, carrying these sometimes to the extent of a *third* or *fourth* of the publication in hand, without any attempt at critical appreciation of its merits or demerits; and he complacently thanks his stars there are none such in France. The refutation of this statement may be safely left to our readers; but we must observe that, did publications such as these really are exist in France, the profession of that country would much benefit by exchanging the present interminable and wearisome original articles, often by obscure writers, (worked up with a display of elementary information, a pedantic magniloquence, and an affectation of definitively settling the matter that would be insufferable here,) which often encumber their journals for prompt, impartial, critical, and copious accounts of every native or foreign original work that appears. Moreover, is not the high position which this writer allows our medical journals assign in their columns to notices of the progress of medicine in France due to the cosmopolitan spirit which has ever actuated the British Medical Press, through whose agency every English practitioner is as familiar with the names and labours of the continental practitioners as he is with those of his own country. Were the same copious notices bestowed by the French medical press upon the various works which issue from this country, the utter ignorance respecting the progress of medical science in England, which at present not only generally prevails among their readers, but too often disgraces their writers, would surely be diminished.

Again it is stated that the "Lectures" published in the weekly periodicals are generally mere reprints of works which have already appeared! We should be curious to see the medical journal that would venture to publish such. Admitting that these lectures must necessarily be very unequal in value, every one knows that several of the courses originally so given to the world, have since been re-published (reversing the order stated by our friend), and now form works of standard and sterling value. The names of Cooper, Abernethy, Clutterbuck, Lawrence, Latham, Liston, Brodie, Murphy, Conolly, Watson, &c. &c. &c., are surely guarantees that the perusal of such lectures will not be without ample profit. In fact, there is scarcely a man of any celebrity in London whose opinions have not been thus published.

The writer is horror-struck at the great number of advertisements, quite unconnected with medicine, which are attached to several of our journals. The practice of advertising, like many others of equal utility, is very little developed in France, and therefore it is no-wise surprising that the medical periodicals do not largely participate in it. In England the advertisements are a separate concern from the professional portion of the journal, and their reception and arrangement does not devolve upon the editor, but upon the publisher—care being always taken that they do not intrench upon the specific space (in conscience large enough for the price) devoted to the proper object of the publication. Advertizers, aware that these publications circulate extensively among medical men, who often have little time for the perusal of those of a more general nature, and believing that the fact of a person pursuing a liberal profession does not exempt him from the ordinary wants of mankind, naturally take the opportunity of offering to his notice whatever they have to dispose of, and what there is reprehensible or ridiculous in this it would be difficult to discover. But we may safely assert, that if the advertisements are more *numerous* in London than in Paris, they are also more *select;* and that even the extra-professional pages of our medical journals will be never found disgraced, as are some of those of Paris, by advertisements of Morrison's Pills, Maison's d'Accouchement, Secret Pills, though approved by Academies; or by woodcuts of quack corsets, and women employing uterine irrigators, &c.

One thing, which is noticed by this writer, we do certainly feel somewhat ashamed of, viz. the eagerness with which young men who have passed a few seasons at Paris display the "slightest honorary grade there acquired, as if it were a most powerful recommendation on the other side of the Channel." We are not disposed to underrate the advantages of a visit to Paris: but we are so far from believing that a *prolonged* residence in that capital is an advantageous preliminary for an English practitioner, that we quite agree with our esteemed predecessor, the late Dr. Johnson, that the first thing persons so educated find on their return is, that they have as much to unlearn as to learn, before they can become safe and efficient practitioners.

---

### Ligature of both Carotid Arteries for a remarkable Erectile Tumour of the Mouth, Face, and Neck. By J. Mason Warren, M.D.

A man, æt. 23, presented himself to the author's notice, the left side of whose face, neck, and mouth was occupied by an enormous erectile tumour of four years' growth. The tongue has been swollen, and the lip occasionally ulcerated, during this period. This last now threatens to take on a cancerous degeneration, and this, together with the fear of fatal hæmorrhage occurring sooner or later, induced Dr. Warren to perform the operation. On the 5*th Oct.*, 1845, the left carotid artery was tied. The patient was about in ten days, the tumour diminishing in size, and the ulcer healing rapidly—his health seeming perfect. *Nov.* 7*th*. The swelling still much diminished and paler. The right carotid was now tied, with no other inconvenience to the patient than drowsiness, and faintness on raising the head. *Nov.* 29*th*. Ulceration of the lip had healed, but as it continned thick and everted by the erectile tissue, it was deemed advisable to remove the diseased portion by a V-like incision, the slight hæmorrhage which attended this operation being easily controlled. *Dec.* 12*th*. The discolouration of the face has become much paler, and the ear resumed its natural size. No pulsation can be felt in the temporal arteries. Just above the clavicle two arteries, nearly the size of the carotids, are seen pulsating powerfully under the skin. ***Feb.* 1*st*.** Patient is in perfect health, and has not had the slightest indications of disturbance in the brain from this interruption of its natural circulation.—*Amer. Journ. Med. Soc., April.*

---

### On the Granular Disease of the Pharynx. By M. Chomel.

During a year or so that M. Chomel's attention has been directed to this affection he has seen, or collected accounts of, about 30 cases. Of these, none has been observed in persons younger than 15. Of 22 cases occurring in his own practice, 17 were males, most of them (like women subject to uterine granulations) being also liable to herpetic eruptions, and especially to acne. In most there is also a peculiar form of the palatine arch, narrowing the nasal fossæ, and contracting the lips, so that these are never completely closed. Such persons sleep with the mouth half open, and awake with it in a very dry state, the pharyngeal follicles becoming, under these circumstances, excessively developed, in consequence of the constant evaporation of the buccal fluids produced by this contact with the air. Thus, also, the persons in whom the complaint is most frequently met with, are those who employ their vocal organs considerably, as orators, singers, advocates, professors, &c.

The affection usually comes on slowly, and excites little uneasiness at first, save from the constant hawking it gives rise to. This sometimes becomes quite sonorous, and is accompanied by involuntary attempts at deglutition, and if the irritation is propagated to the œsophagus, a frequent desire to drink. There is a dryness or itching in the pharynx, and the voice is more or less affected. The expectoration consists of transparent viscous globules of a slight opaque tinge, or streaked black or slate colour. On examination, we find the pharynx covered with little red points the size of hemp-seed, but sometimes being much more voluminous. They may be grouped into arabesque-like forms, into little discs, or as nipple-shaped projections. Granules are also usually found upon the palate and uvula, but then they are more discrete. The mucous membrane retains its normal characters.

The course of the disease is always chronic and remitting, it being especially troublesome in cold and damp weather. It never spontaneously disappears, and often obstinately resists treatment. Of 14 cases which M. C. had occasion to see at more or less remote periods after treatment, but 4 remained cured, the others being only relieved. The diagnosis of the affection is easy enough, as it obviously consists in hypertrophy of the numerous muciparous follicles of the pharynx. In *treating* the disease, M. Chomel has tried all descriptions of astringent gargles, &c. with but indifferent success, and when it proves obstinate, cauterizing the mucous membrane is the only means likely to prove serviceable, fluid caustics having been found more useful in his hands than solid ones.—*Gazette Medicale,* No. 16.

---

### On Paracentesis Thoracis in Acute Pleuritic Effusion. By M. Bricheteau.

In reporting upon an additional account of this operation, recently presented to the *Academie* by M. Trousseau, M. Bricheteau adverts to the fluctuations of opinion which have occured in reference to it, as applied to empyema, in both ancient and modern times. It was in 1841 M. Trousseau adduced two or three successful instances of its application to cases of recent pleuritic effusion, and in the present paper another is brought forward. The patient, æt. 24, had suffered from pleurisy about 20 days prior to admission. The side was notably dilated, and the effusion not yielding, a puncture was practised between the 7th and 8th ribs, and about 70 oz. of a clear fluid discharged with manifest relief, the patient being dismissed in about a fortnight afterwards, She has been since seen by M. Bricheteau, who found her cure confirmed. Two other successful cases have occurred since this paper was presented, the particulars of which are furnished by M. Bricheteau.

In considering whether M. Trousseau's success is such as to warrant the recommendation of the imitation of his practice by the *Academie,* M. B. observes, we must bear in mind that, even in the last century, when our diagnosis was so much more uncertain, examples of cure by this means were by no means rare. MM. Monneret and Fleury state that, of 66 instances in which we have accounts of its having been performed, in 56 the effusion was the result of pleuritic inflammation, and that of these 42 were cured and 14 died. M. Trousseau believes that we should not wait until the dyspnœa become very urgent and suffocation impending, as danger may exist, if the fluid have gradually accumulated, long before this. If the dulness upon percussion be perceived along the median line, as low as the 4th rib, the puncture may be made, although the dyspnœa be not urgent; while, if it extend daily across the median line, towards the opposite side, the necessity for interfering is still more apparent, especially if the effusion be on the left side, and tends to displace the heart towards the right. If, even when the dulness does not extend beyond the median line, there is orthopnœa, a small, frequent pulse, anxious countenance, and especially any tendency to lithopymia, we should operate at once.

M. Trousseau prefers evacuating all the fluid at one operation, and guards against the admission of air (by disposing a piece of gold-beaters' skin as a valve over the orifice of the canula), which might oppose the re-distension of the pulmonary structure in its now enlarged cavity. The difficulty with which a condensed lung re-expands is far less in recent than in chronic pleuritic effusion, as firm adhesions have not formed, and the elasticity of its texture is not yet destroyed. In M. T's cases, the Reporter ascertained that the air penetrated immediately into every portion of the pulmonary structure. The existence of pulmonary tubercle is not an absolute objection to the operation, as the removal of fluid in this case has sometimes at least prolonged life.—*Gaz. Med. Chir.* Nos. 17 and 18.

(In the brief discussion which followed, M. *Louis* observed that, he thought the operation should be more cautiously recommended. He had seen many cases of pleurisy, but none in which it appeared indicated; nor had he ever met with a case which terminated fatally when the disease was in its simple form. M. *Rochoux* stated that, although cases in which this operation could be called for must be very rare, that yet they may occur, and that more frequently in acute than chronic pleurisy. M. *Bricheteau* agreed in this last opinion. We believe with the above practitioners that, the necessity of an operation in simple pleurisy, properly treated, can never hardly occur: but the case is very different in more complicated instances, and in even simple ones which have been neglected. In such we doubt not of its propriety, and see no reason to hesitate about its performance. The extension of the operation has in this country met with many advocates of late, and we may refer among others to the papers of Drs. Hughes and Roe, in the Guy's Hosp. Rep. and Medico-Chir. Trans.—See Med. Chir. Rev., N. S. Vol. I.—*Rev.*)

---

### Management of the Puerperal State.

In M. *Piorry's* Ward at *La Pitié* are women suffering from fever producing eschars on the sacrum, and others brought from the *Maternité* labouring under puerperal peritonitis, and yet, although the ceiling is low and the beds crowded, no instance of phlebitis, phlegmasia dolens, or peritonitis, putting on the dangerous character so common in the puerperal state, has ever been observed among the women delivered therein during the ten years he has had charge of it. In this same ward five women have been brought from a *maison d'accouchement* suffering from utero-peritonitis, and of these three died. At the *Maternité* the mortality has been such as to lead to the distribution of the remaining patients among the other hospitals. M. Piorry, regarding any ill-effects which may result from the admission of cold air as minor evils, has all the windows, however near the beds, kept wide open and the bed-curtains drawn back, taking care, however, that the woman's exposed person do not become subjected to the currents. Immediately after delivery, he orders the vagina to be carefully injected with tepid water every hour or two. He has the abdomen rubbed with olive oil, orders good diet, and keeps the bowels open with aperients and enemata. He thinks lying-in women should be admitted into the general hospitals, and not congregated together in special ones.—*Gaz. des Hôpitaux*, No. 43.

---

### On the Public Health of Paris during the first Quarter of 1846.

The *Gazette Medicale* has recently published some interesting articles upon this point. From the Registries of the Paris Observatory, it appears that—1. The *temperature* was unusually high. 2. The *atmospheric pressure* more considerable than ordinary. 3. A remarkably small quantity of rain fell in February. 4. South and west winds predominated. 5. The oscillations of the thermometer and barometer were neither considerable or sudden. 6. All the above characteristics were more marked in February than in the other two months.

The *diseases* of the quarter may be considered in reference to their *form*, *severity* and *frequency*. 1. As regards the *form*, the most remarkable thing was the *rarity of the acute affections of the respiratory organs usually met with in winter*—bronchial catarrh itself being even far from common. Even those which did present themselves were very mild, sometimes assuming rather a neuralgic than an inflammatory character. The diseases *commonly met with in summer and autumn* were on the other hand frequent. Thus *gastric* and *bilious fevers* were very prevalent, and what is still more uncommon at this season several cases of *typhoid fever*, generally of an ataxic form occurred. Others of a rare disease at Paris, *remittent fever* were met with, the adynamic form prevailing. In February and March *puerperal fevers* abounded, women who were not pregnant or puerperal, and others who had been confined for a fortnight or so, suffering at the same period from induration of the cellular tissue of the pelvis, and pains in the renal and abdominal regions. Finally, a malignant and fatal form of *measles* prevailed. *Adynamia and ataxia* formed the common character of these various affections. In fact, the season of the quarter from January to April resembled that of summer and autumn combined. To a very unusual and little changing elevation of temperature, were conjoined on the one hand southerly and westerly winds, and on the other, in January and March considerable falls of rain ; and the simultaneous inversion of the medical and atmospheric constitutions is remarkable. Medical constitutions do not, however, depend upon the absolute quantity of heat or moisture; and a given atmospheric condition will produce different effects according to the epoch of the year at which it is observed. Thus, the temperature during which these affections appeared was far less elevated than that which is observed at the time of their prevalence in summer.

2. The *frequency* of disease, as exhibited by the number of admissions into the hospitals was increased ; for, while these amounted to but 18,807 in the first quarter of 1845, when the weather was much colder from northerly winds, and the atmospheric perturbations much more considerable, they reached 19,846 in the quarter of 1846. *Diseases put on different forms then, but increased in number*. In examining their distribution over different months, one is struck with the comparative immunity of February, during which there were 300 patients less than in January, and 850 less than in March. Of all the atmospheric conditions the *quantity of rain* alone considerably differed in this month. The total quantity of rain which fell during the first quarter of 1845 was less considerable than that which fell during the same period of 1846 in the proportions of 12·060 to 15·067. In both years, too, the amount which fell in February was considerably less than that which fell in the other two months of the quarter, and the numbers of admissions diminished in like proportion.

3. The *severity* of the diseases can only be imperfectly estimated by the number of discharges from the hospitals. From these, however, their benign character may to some extent be inferred—since of a hospital population of 37,056 in the quarter of 1846, there were 18,213 discharges ; while of a population of 36,043 in 1845, there were but 16,695. The generally mild character of prevalent affections does not however necessarily imply a *small proportion of deaths*. In this ataxic constitution the majority of the patients do not exhibit alarming symptoms, but amid apparent security diseases from time to time put on unusually and unexpected fatal characters. Thus, in spite of the large number of dismissals that of the deaths was yet greater than in 1845. In that year, of 36,043 patients 1958 (1 in 18·4) died, while the deaths of 1846 were 2073 in 37,056 patients (1 in 18·1).—*Gazette Medicale*, Nos. 18, 19, 20.

(It may be interesting to compare some of these *data* with those furnished by the Registrar-General's First Quarterly Report for 1846. By this we find that the mean temperature at Greenwich was nearly 5° Fah. above the average of 25 years, and 8° above that of the first quarter of 1845. South-west winds prevailed, and the fall of rain was as high as 5·73 at Greenwich, it being but 4·80 in 1845. With these meteorological conditions the deaths in a population of 6,579,653, amounted to 43,708, being less by 6166 than those (49·874) registered during the same quarter of 1845.

Confining our attention to the *metropolis* (1,915,104 souls), we find that the deaths from all causes registered in the quarter ending with March 1846 (12,376), were less by 2152 than those registered (14,528) for the same quarter of 1845. Notwithstanding this gross result the following diseases *exceeded* the average mortality of the winter quarters of the seven preceding years of registration. *Measles* (viz. 401 deaths instead of 273), *Hooping-Cough* (as 767 to 504), *Diarrhœa* (as 119 to 72), *Bronchitis* (as 758 to 320), and *Rheumatism* (as 62 to 35). The mortality of the following has diminished—*Small-pox* (as 77 to 291), *Scarlatina* (as 221 to 357), *Croup* (as 79 to 107), *Convulsions* (as 511 to 727,) and *Pneumonia* (as 946 to 1178). Taking the Endemic, Epidemic and Contagious diseases *en masse*, the mortality is very near the average (as 2222 to 2277), which is the case also with regard to typhus (410 to 403), and deaths from *Childbed* (101 to 104.)

It will be seen that the statement of the influence of rain in augmenting the amount of sickness and mortality alluded to in the French Report is not verified in that of the English one; nor have precisely the same diseases especially raged in London as in Paris. It is true we are here only speaking of deaths and not also of unfatal diseases; but every practitioner must know, to his cost, how much less numerous these latter have been during this quarter than usual; and most will have remarked the adynamic and neuralgic characters of many diseases not usually so distinguished.—*Rev.*)

---

### On the Excessive Mortality of Male Children.
### By Dr. Emerson.

Dr. Emerson observes that, ever since correct mortality registers have been kept, a larger proportion of males have been found to die in the early years of life than females. In Europe, the male *births* exceed the female ones by about 5 or 6 per cent., and in Philadelphia by more than 7½ per cent., and yet the number of boys and girls are found very nearly equalized by the 10th year, while by the 15th, the females outnumber the males almost as much as the latter did the former at the time of birth. In Philadelphia the deaths of boys to the 15th year exceed those of girls by about 15 per cent.

Upon examining the question more in detail, Dr. E. found that *inflammation of the brain* and its consequences, *convulsions, hydrocephalus,* and *inflammations* in general, were the diseases which especially proved fatal to male children, while *pertussis, scarlatina* and *phthisis* were those which proved generally fatal to female children. That is to say, that the males perish from the *sthenic,* the females from the *asthenic,* class of affections. On examining the London Tables he found the same disposition nearly prevailed, and the practical deduction he draws from the whole is, that the treatment of the diseases of males should be more prompt and vigorous than that of those of females.

The result of his observations upon *temperature* leads him to conclude that the hot months in America act as injuriously upon infantile life as do the winter ones; but that, after the child has weathered the first few months of its existence, neither the one or the other seems to especially determine mortality.—*American Journ. Med. Sciences.*

### MIALHE ON THE ASSIMILATION OF AMYLACEOUS AND SACCHARINE SUBSTANCES.

A Report has just been made to the Academy upon the continuation of M. Mialhe's researches upon this subject. He observes that it has been long acknowledged that *albuminous* or *azotized* aliments are digested through the agency of *pepsine* which is a true ferment, and *fatty* bodies by that of the bile; but that it has been proved only by his own researches that *amylaceous* and *saccharine matters* are assimilated through an active principle of the saliva. This principle exerts no action upon azotized substances, fibrin, albumen, &c.; but it exerts a most remarkable one upon starch, especially when this is completely disintegrated by bruising, heat, water, &c. One part by weight suffices to liquify and convert into dextrine and sugar more than 2000 of fecula. This transformation is not without its analogy in science, for there exists a body which exerts upon starch a specific power exactly resembling that of the salivary ferment, viz. *diastase* or the active principle of germinated barley, discovered by MM. Payen and Persoz. A long course of experiments lead to the inference that the two principles are identical, although M. M. contemplates performing others for the further elucidation of this point. In the mean time, he proposes terming the active principle of the saliva of man *animal diastase* or *salivaire,* in distinction of that of grain which may be termed *vegetable diastase.* The proportion of each existing in saliva and barley respectively, is 2000 parts. To obtain it, saliva should be treated with 5 or 6 times its weight of pure alcohol, adding this as long as any precipitate is produced. The diastasis is deposited in little white flakes, which are to be collected on a filter and dried between two thin plates of glass in a current of air at a temperature of from 104° to 122°, and kept in a well-corked phial. It is a white or greyish, amorphous solid, insoluble in alcohol, but soluble in water and in diluted alcohol.

The following are the conclusions. 1. The saccharifaction of starchy matters is effected by the influence of the diastase which exists in the normal state in the liquid secreted by the pancreatic and salivary glands. 2. This transformation of amylaceous substances into dextrine and glucose is not as some believed a pathological but a physiological fact; for without it they would cease to be alimentary, not being indeed absorbable until acted upon by the diastase. 3. The dextrine and glucose (*i. e.* the newly saccharified matter) to become assimilated *must be transformed by the alkalis of the blood* into new products, the chief of which probably are the kali-saccharic acid, formic acid, and ulmin. 4. If the alkalescence of the blood is insufficient (the blood having become too feebly alkaline, neutral, or even acid), the transformation will not take plaee; the sugar becomes a foreign body in the economy, and as such is excreted by the kidneys, giving rise to *diabetes* or *glucosuria.* 5. Saccharoid bodies play an important part in the great act of nutrition, and do not serve merely as materials for respiration, as some eminent men have supposed; but on the contrary it is certain they participate in the chemico-vital re-actions which preside over the incessant organic mutations, and it results that, if their assimilation is abolished (chronic diabetes), or simply vitiated (acute diabetes), the anormal molecular decompositions are effected at the expense of the living tissues and liquids, and it is hence we observe—1. A general disturbance of the humours of the economy from defect of alkalescency, which gives rise to feebleness of sight, capillary engorgements, and pulmonary consumption. 2. A profound alteration in nutrition, leading to debility, languor, and wasting. It is to these two physiologico-pathological facts we must attribute the constantly fatal termination of diabetic diseases, when by a methodical plan of treatment the power of decomposing the amylaceous class of aliments has not been restored to the chemical laboratory of the human body.

According to these views, M. Mialhe recommends the employment in the treatment of diabetes of an animalized regimen, as little fecula as possible, and the use of the alkaline bases and their carbonates, as magnesia and lime. The number of cases improving under such a plan are not sufficient to induce the *Acadamie* to pronounce definitively upon this point; although it entertains a high opinion of the value of M. Mialhe's researches, and begs him to continue them. *Comptes Rendus,* No. 12, and *Gazette Medicale,* Nos. 18 and 19.

---

### Varia.

*Thoracic Vibration.*—M. C. Broussais observed that he is surprised this sign is so much neglected in practice. In health it is much less marked in persons having weak or sharp voices, and in whom the cellular tissue is infiltrated or adipose, than in those whose voices are grave, and the walls of whose chests are of little thickness and only covered by their muscular layers. In disease it becomes *diminished,* in pleuritic effusion (and perhaps in the case of false membranes) re-appearing when the fluid is absorbed. It is *increased* in the induration of the parenchyma by red or grey hepatization, in chronic pneumonia, and when the tissue of the lung becomes condensed by pleuritic effusion, providing this be not too abundant, when the lung becomes reduced to nothing, or too sparing, when it is not sufficiently condensed.—*Gaz. des Hôpitaux,* No. 46.

*Rheumatic Carditis.*—M. Chomel, in a recent clinical lecture, stated that he believed that affections of the heart as a consequence of rheumatism, are of exceeding rare occurrence.—*Gaz. Med. Chir.,* No. 13.

*Extemporaneous Vesication for Endermic Medication.*—Place a piece of silver money on a plate, and lay upon it two circles of old linen of rather less diameter. Saturate these with *liq. ammon.* and then apply the apparatus, with its linen surface downwards, to the skin, and maintain an equable pressure with the finger. In 10 minutes the skin is observed to redden at the circumference of the money, and on the apparatus being removed vesication will take place.—*Bulletin de Therapeutique.*

*Compression of the Carotid in Epistaxis.*—Two cases are related in which obstinate epistaxis was effectually arrested by temporary compression of the common carotid artery.—*Gaz. Med. Chir.* No. 23.

*Endermic Use of Iodine.*—Dr. Diver recommends the following formula as an excellent application in scrofulous enlargement of the glands of the neck. ℞. *Iodin.* 1 p, *Bals. Canad.* 3 p, *Picis. Abietis* 3 p. Triturate the Iod. with the B. C., and melt the pitch at a gentle heat, mixing the whole together just as it is about to cool. Spread on kid for immediate use.—*Phil. Med. Exam.*

*Quinine in Rheumatism.*—In addition to M. *Briquet's* testimony in favour of large doses of Quinine in rheumatism, quoted at p. 267, we may cite that of Dr. Dunglisson, who looks upon it as a powerful sedative, capable of promptly curing the great majority of cases. He gives it fearlessly in every case, never having seen any ill-effects result, beginning with 18 or 20 grs. in the 24 hours, and gradually increasing the quantity.—*Philadelphia Medical Examiner.*

*Local Paralysis from Pressure.*—A woman under M. Guerard's care has complete paralysis of the fore-arm and hand from using crutches for a fortnight. During the few days the patient has kept her bed the fingers have somewhat regained their motility (*Gaz. des Hop.*) M. *Pigeolet* relates the case of a person who having slept during a journey with his arm compressed between his head and the edge of a carriage, it became paralyzed for six weeks. Every kind of counter-irritant was unavailingly tried until an embrocation, composed of *ol. oliv.* and a few drops of *tr. lyttæ,* was prescribed, which was speedily successful. An Italian surgeon has also of late met with several cases in which paralysis has been induced by laying upon the arm in bed.—*Gaz. Med.*

*** The intimation, which we have given at page 230, will account for our omission of any extracts from the French Journals respecting the recent very important discussion on *Plague* and *Quarantine,* in the French Academy.

---

